# Host Immune Response to Intestinal Amebiasis

**DOI:** 10.1371/journal.ppat.1003489

**Published:** 2013-08-22

**Authors:** Shannon N. Moonah, Nona M. Jiang, William A. Petri

**Affiliations:** Division of Infectious Diseases and International Health, Department of Medicine, University of Virginia Health System, Charlottesville, Virginia, United States of America; University of Wisconsin Medical School, United States of America

## Introduction

**Figure 1:**
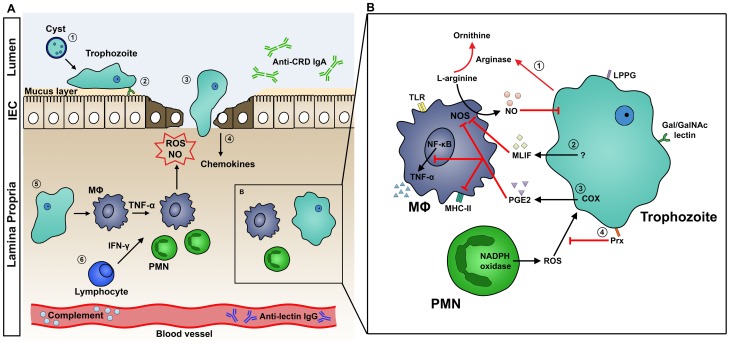
A. Host Immune Response to Intestinal Amebiasis. (1) Stomach acid serves as a first line of defense against enteropathogens, but amebic cysts are highly resistant and excyst in the lumen of the intestine. (2) Mucin, a glycoprotein secreted by goblet cells and submucosal glands, is the main constituent of the protective mucus layer. Trophozoites attach to the host tissue surface via Gal/GalNAc lectin. (3) Amebae secrete cysteine proteases, which disrupt the mucus layer and facilitate tissue invasion. (4) Injured IECs release potent chemokines to recruit immune cells to the site of invasion. (5) Activated macrophages release TNF-α, stimulating PMNs and macrophages to release ROS and NO, which kill the parasite. ROS and NO may also contribute to tissue destruction. (6) IFN-γ released by lymphocytes activates macrophages and PMNs. B. Mechanisms of Host Immune Evasion. (1) *E. histolytica* trophozoites inhibit the respiratory burst of MΦ using arginase, which converts L-arginine, a substrate of NOS, to L-ornithine. This depletes the L-arginine supply that macrophages use to produce NO. (2) MLIF produced by ameba suppresses NO production. (3) COX in ameba or ameba-exposed macrophages produces the immunoregulatory molecule PGE2. PGE2 suppresses macrophage effector functions by elevating cAMP levels, which in turn inhibits NO production, MHC-II expression, and TNF-α production. (4) Amebic Prx, a 29-kDa surface protein, confers resistance to neutrophil reactive oxygen species. Abbreviations: COX, cyclooxygenase; IEC, intestinal epithelial cells; IFN-γ, interferon-gamma; MΦ, macrophage; MHC-II, major histocompatibility complex class 2; MLIF, monocyte locomotion factor; NO, nitric oxide; NOS, nitric oxide synthase; PGE2, prostaglandin E2; PMN, polymorphonuclear leukocytes; Prx, peroxiredoxin; ROS, reactive oxygen species; TNF-α, tumor necrosis factor-alpha.

*Entamoeba histolytica* is an invasive enteric protozoan parasite that causes amebiasis. Globally, diarrheal disease is second only to pneumonia as a leading cause of death in children under five, and intestinal amebiasis is one of the top ten causes of severe diarrhea in the developing world. Amebiasis is more common in malnourished children, a state that afflicts approximately one-third of children in the developing world. In the critical first year of life, 11% of Bangladeshi infants living in poverty suffer from *E. histolytica* diarrhea [1], [2]. There is currently no vaccine for this devastating disease, thus an understanding of the human immune response toward the parasite would greatly enhance the ability to develop effective immunotherapies. The host deploys a series of immune defenses against the parasite as it invades the colon. The ameba, however, has developed complex strategies to evade host defenses and promote its own survival. Here, we summarize the dynamics of the interaction of parasite with host and its importance in the pathogenesis of amebiasis (Figure 1).

## Innate Immunity

Stomach acid serves as an important first line of defense against enteropathogens through its ability to kill acid-sensitive microorganisms. However, infectious amebic cysts are highly resistant and survive passage through the acidic environment of the stomach. In the intestine, the next layer of innate defense may be the mucus layer, which is thought to act as a protective barrier, preventing *E. histolytica* from invading intestinal epithelial cells (IECs). Mucin, a major constituent of the intestinal mucus layer, is a glycoprotein secreted by goblet cells and submucosal glands. Mucin glycoproteins bind to and inhibit the Gal/GalNAc adherence lectin of the parasite, preventing in vitro adherence and killing of CHO cells [3]. Trophozoites, however, can disrupt the mucus layer and intestinal barrier by secreting cysteine proteases (CPs) and glycosidases to allow for penetration of the colonic mucosa. Specifically, *E. histolytica* cysteine protease-A5 (EhCP-A5) degrades mucin-2 (MUC2) and extracellular matrix (ECM) proteins [4]. The importance of cysteine proteases was demonstrated by an ex vivo human intestinal model, where EhCP-A5–silenced parasites failed to penetrate into the colonic lamina propria [5].

IECs exposed to *E. histolytica* trophozoites secrete potent chemokines, such as IL-8, resulting in immune cell recruitment and infiltration of the lamina propria and intestinal epithelium [6]. Neutrophils are one of the first immune cells to respond to amebic invasion. Neutrophils activated by interferon-γ (IFN-γ), tumor necrosis factor-α (TNF-α), or lipopolysaccharides (LPS) carry out amebicidal activity in vitro by releasing reactive oxygen species (ROS) [7], [8]. Depletion of neutrophils with anti-Gr-1 antibodies resulted in exacerbated intestinal disease in murine models, supporting the protective role of neutrophils in amebiasis [9]. It should be noted, however, that anti-Gr-1 antibodies can deplete other granulocytes such as eosinophils.

Macrophages also play a crucial role in the host response against intestinal amebiasis. Macrophages are amebicidal after stimulation with IFN-γ or TNF-α [10], [11]. Several amebic antigens are known to activate these cells via pattern recognition receptors. Toll-like receptor (TLR)-2 expression in macrophages is upregulated when exposed to the Gal/GalNAc lectin of *E. histolytica*, triggering pro-inflammatory cytokine production via NF-κB activation [12]. Macrophages that lack TLR-2 and TLR-4 displayed impaired response to *E. histolytica* lipopeptidophosphoglycan (LPPG), suggesting that pattern recognition is essential to the immune response [13]. Additionally, *E. histolytica* DNA can activate macrophages through interacting with TLR-9 [14]. Amebicidal activity of macrophages is contributed to by the production of nitric oxide (NO) from L-arginine, which is mediated by macrophage nitric oxide synthase. Inducible nitric oxide synthase (iNOS)–deficient mice were more susceptible to amebic liver abscess and to *E. histolytica*–induced hepatocytic apoptosis, implicating a critical role for NO in the host defense against amebiasis [15].

## Adaptive Immunity: Mucosal Immunoglobulin A and Cell-Mediated Response

The Gal/GalNAc lectin is the major amebic surface adhesion molecule and mediates binding to the colonic mucus layer as well as carbohydrate determinants on a variety of host cells including epithelial cells. The heavy chain of Gal/GalNAc lectin contains the carbohydrate recognition domain (CRD) that is responsible for binding. In both mice and baboons vaccinated against *E. histolytica*, IgA antibodies against Gal/GalNAc lectin correlated with protection [16], [17]. In a cohort of preschool children in Dhaka, Bangladesh, mucosal IgA directed at the CRD domain was associated with protection of children from *E. histolytica* infection and disease. Conversely, serum anti-lectin IgG was not associated with protection, but instead was associated with an increased frequency of new *E. histolytica* infections [18].

Cell-mediated interferon gamma (IFN-γ) appears to provide protection from amebiasis through its ability to activate neutrophils and macrophages to kill the parasite. In a prospective study, children's peripheral blood mononuclear cells (PMBCs) were stimulated with soluble amebic extract and IFN-γ levels were measured. Children with higher IFN-γ production had a significantly lower incidence of future *E. histolytica* diarrhea [19]. This finding was supported by murine vaccination studies, which showed that vaccine-induced protection against *E. histolytica* infection could be passively transferred to naïve animals by IFN-γ–producing T cells. In addition to IFN-γ, IL-17 was also shown to contribute to vaccine-induced protection in murine studies [20], [21]. These findings suggest an important role for cell-mediated cytokine production in protection from amebiasis.

## Mechanisms of Parasite Evasion

Although the host deploys a robust immune response against *E. histolytica*, the parasite has developed a remarkable number of mechanisms to evade these attacks (Figure 1B). While neutrophils are capable of killing ameba, virulent ameba are far more effective at lysing and phagocytosing neutrophils: in vitro, one trophozoite can kill upwards of 3,000 neutrophils [8]. In addition, amebic peroxiredoxin, a 29-kDa surface protein with antioxidant properties, protects the parasite from neutrophil reactive oxygen defenses [22]. *E. histolytica* has also developed several strategies to modulate macrophage responses. Amebic arginase converts L-arginine, a substrate of macrophage NOS, to L-ornithine, thereby limiting NO production by macrophages [23]. Cyclooxygenase (COX) in amebae can produce prostaglandin E2 (PGE2), which elevates the cyclic adenosine monophosphate (cAMP) levels in macrophages [24]. This, in turn, inhibits protein kinase C (PKC)–mediated expression of class II MHC [25]. Furthermore, monocyte locomotion inhibitory factor (MLIF) has been implicated to modulate macrophage function by inhibiting NO [26].

Complement may act to prevent dissemination of trophozoites and extraintestinal disease. Once amebae activate the complement system, membrane attack complexes (MACs) form and lyse the parasites. The parasite has at least two mechanisms to resist complement. Gal/GalNAc lectin, which shares sequence similarity and antigenic cross-reactivity with the MAC-inhibitory protein CD59, inhibits the formation of the C5b-9 complex, and thus prevents lysis by MACs [27]. Additionally, CPs can cleave complement factors [28].

## Host Inflammatory Response Contributes to Tissue Damage

An appropriate immune response clears pathogens without causing significant damage to the host tissue. Tissue destruction in amebic colitis arises from both *E. histolytica* cytolytic factors and the resultant gut inflammatory response. While TNF-α stimulates neutrophils and macrophages to release ROS and NO to fight the parasite, an excess amount of TNF-α can result in direct damage to host tissue.

Higher TNF-α production was recently shown to correlate with *E. histolytica* diarrhea in children. Each 1,000 pg/mL increase of TNF-α correlated with an 18% increased chance of acquiring *E. histolytica* diarrhea [29]. Blocking TNF-α with monoclonal antibodies reduced inflammation and intestinal damage in amebic infection in the severe combined immunodeficient mouse-human intestinal xenograft (SCID-HU-INT) model [30].

The anti-inflammatory cytokine, interleukin-10 (IL-10), is an important immunoregulator in the intestinal tract. IL-10 counteracts an exaggerated pro-inflammatory immune response by inhibiting the production of inflammatory mediators such as TNF-α. Disruption of the IL-10 gene in mice results in colitis and is used as a model to study inflammatory bowel disease. Additionally, in a phase I trial, transgenic bacteria expressing IL-10 were shown to decrease disease activity in Crohn's patients [31]. IL-10–deficient C57BL/6 mice are susceptible to amebic colitis, supporting the role of inflammation in *E. histolytica* pathogenesis [20], [32].

## Host Genetics, Immunity, and Susceptibility to Amebiasis

Millions of people worldwide are colonized with *E. histolytica*, yet only 20% develop symptomatic disease, with highly variable outcomes [33]. Host genetic makeup may explain, in part, why individuals differ in their susceptibility to amebic infection. Leptin, an adipocytokine first characterized for its metabolic effects, is now recognized as an important modulator of the immune system. Normal leptin signaling may mediate resistance to amebiasis via several mechanisms such as stimulating a Th1 response, inducing anti-apoptotic pathways, and promoting tissue repair. In a murine model, leptin-deficient (*ob/ob*) and leptin receptor–deficient (*db/db*) mice were highly susceptible to *E. histolytica* infection, while wild-type C57BL/6 mice were resistant [34]. In a nine year prospective study in a cohort of preschool children in Dhaka, Bangladesh, a single amino acid polymorphism (Q223R) in the leptin receptor was associated with increased susceptibility to *E. histolytica* infection. Children with two arginine alleles (223R) were nearly four times more likely to suffer *E. histolytica* infection as compared to those homozygous for glutamine (223Q). Similarly, mice with at least one 223R allele were significantly more susceptible to amebic infection and exhibited greater levels of intestinal epithelial apoptosis and mucosal destruction following infection [35]. The site of leptin-mediated resistance was localized to the intestinal epithelial cells by tissue-specific knockout. An in vitro model showed that leptin signaling protected human epithelial cells from amebic killing via a STAT3-dependent pathway [36].

Human leukocyte antigens (HLAs) play a crucial role in the immune response and are also highly polymorphic. The ability of specific HLA II alleles to present amebic antigens to CD4+ T cells may alter susceptibility to *E. histolytica*. It was found that children who were heterozygous for the HLA class II DQB1*0601/DRB1*1501 haplotype were more likely to be *E. histolytica* negative [37].

## Future Perspectives

Several decades of research have led to an improved understanding of the host immune response to intestinal amebiasis. Despite these advances, fundamental questions remain unanswered. For example, the immune mechanism that explains why only a subset of exposed individuals develops clinical disease is not fully understood. This is arguably one of the most important knowledge gaps to be filled. Additionally, the emerging field of microbiome science opens new avenues for amebic research. The effect of the microbiota on the immune response to *E. histolytica* and/or its virulence is not yet known. The answers to these questions may lay the foundation for developing an effective vaccine against this devastating disease.
